# The Rates of Tax

**Published:** 1919-10-04

**Authors:** John Burns


### INCOME-TAX LIABILITIES AND CLAIMS.

### II. The Rates of Tax.

By JOHN BURNS, W.S., Edinburgh.

As is well known, the rate of tax varies according as the income is earned or unearned and according to its total amount. The amount of the income is ascertained by taking all the sources of income, adding them together, including wife's income, and from the total taking off such deductions as ground burdens, mortgage interest, bank interest, interest on unpaid price of practice, annuities, and any other similar outgoings. Thus take this case.

Gross Income.

| | | |
|---|---|---|
| Profits of practice on the three years' average ... | | £700 |
| Salary from appointment, the year's amount ... | | 100 |
| From investments ... ... ... ... ... | | 100 |
| Wife's income from investments ... ... ... | | 150 |
| Residence, property of self or wife, entered at the net Schedule A assessment ... ... ... | | 50 |
| | | £1,100 |
| *Deductions.* | | |
| Ground burdens ... ... ... ... ... | £10 | |
| Mortgage interest ... ... ... ... ... | 40 | |
| Interest on unpaid price of practice ... | 60 | |
| Annuity to partner's widow ... ... | 300 | |
| | | £410 |
| *Note.*—It is recognised that these deductions in themselves are not very happy, but they bring out the income-tax point. | | £690 |
| *Income-tax Abatements.* | | |
| Scale, small income ... ... ... | £70 | |
| Wife ... ... ... ... ... ... | 25 | |
| Four children under sixteen (£25 each) | 100 | |
| Incapacitated dependent relative ... | 25 | |
| Life insurance premiums ... ... | 70 | |
| | | £290 |
| | | £400 |

Now here we have three figures—£1,100, £690, and £400. Which of these is to determine the rates of tax and the right to abatements? The answer is the £690. The reason is that that is the "statutory" net income of the taxpayer. The £1,100 does not answer that description, for it includes the four items of ground burdens, etc., amounting to £410, which are income, not of the doctor at all, but of other parties—namely, the creditors who receive them, including the ex-partner's widow. Nor can the final £400 be the doctor's "income," for it is arrived at by deducting items which do not reduce his income at all, as, for instance, the statutory fixed scale abatement of £70, to which he is entitled because his "income" does not exceed £700. This illustration shows very completely the standard by which rate liabilities and abatement rights are fixed.

The next thing is to see what the rates are. We go back to April 1916 because repayment claims are still allowed back to that date.

| Not exceeding* | Years to April 5, 1917 and 1918. Rate on Part which is Earned | Unearned | Year to April 5, 1919 and 1920. Rate on Part which is Earned | Unearned |
|---|---|---|---|---|
| £ | s. d. | s. d. | s. d. | s. d. |
| 500 | 2 3 | 3 0 | 2 3 | 3 0 |
| 1,000 | 2 6 | 3 6 | 3 0 | 3 9 |
| 1,500 | 3 0 | 4 0 | 3 9 | 4 6 |
| 2,000 | 3 8 | 4 6 | 4 6 | 5 3 |
| 2,500 | 4 4 | 5 0 | 5 3 | 6 0 |
| over 2,500 | 5 0 | 5 0 | 6 0 | 6 0 |

* Total income, earned and unearned, including wife's.

Most incomes are partly earned and partly unearned. That just means that the earned part pays the one rate, and the unearned part the other rate.

Income tax is partly collected "at the source"—that is, by deduction before you receive the income, and partly by direct assessment by the Inland Revenue. When it is collected in the latter way it is charged at once at the proper rate according to your total income, assuming that the proper return has been made to enable the authorities to do so. But when it is taken off at the source—*e.g.* from dividends—it is stated at the highest rate (at present 6s.), and then this has to be adjusted in some way so as to reduce it to your true liability, if the total income does not exceed £2,000. This adjustment may require a repayment claim, but also it may be accomplished by an allowance from any direct assessment—*e.g.* on profits; or on your residence, or on War Loans or Bonds (not bearer). Thus, to take a simple case, suppose an income of £2,000, half from the practice, and half from the doctor's and his wife's investments, these latter all being charged at the source at 6s. Now the true rates are 5s. 3d. on these and 4s. 6d. on the earned £1,000. In assessing the earned £1,000 the rate would be taken at once at 4s. 6d., which would show a tax amounting to £225, off which, however, there would be allowed a rectifying 9d. per £ on the £1,000 from investments, equal to £37 10s. And so the net charge would be £187 10s., which would just put everything right.

War Service Rates.

It is, we think, common knowledge that there is a specially low rate of tax on those engaged in the war. This includes the R.A.M.C. How far it goes is at present under consideration. There is the question whether it includes lady doctors in charge of military hospitals, and we know that it has been refused to some medical men in the charge of such hospitals, but doing civil practice also, holding no commission, wearing no uniform, and although the pay is drawn through military channels. It is thought that there is a real grievance here, but though the question has been agitated both with the Departments and by Sir Watson Cheyne in Parliament, the lower rate is still refused in these cases. Rightly or wrongly, it is inferred that the War Office is sympathetic and that the real difficulty lies in the Inland Revenue Department. What is said by the Department is that one of the doctors interested ought to contest the point before the local commissioners. He can do so without legal assistance; it is not unlikely that he would find the tribunal favourably inclined, and if he detained their decision in his favour it is quite possible that the Department might not contest the point further.

In any case note that when this privilege of low rate of tax does apply, it holds with reference only to *the pay*, but that it does apply to the pay no matter how large may be the total income. The special rate is—

| If Total Income does not exceed | Rate on Pay | If Total Income does not exceed | Rate on Pay |
|---|---|---|---|
| £ | s. d. | £ | s. d. |
| 300 | 0 9 | 2,000 | 2 9 |
| 500 | 1 3 | 2,500 | 3 3 |
| 1,000 | 1 9 | over 2,500 | 3 6 |
| 1,500 | 2 3 | | |

The previous article appeared on August 30, p. 541.

**Income-Tax Liabilities and Claims**—*(continued)*.

Also the following additional rights attach—namely (1) a deduction for uniform when worn; (2) exemption limit of £160 instead of £130, which cannot interest our readers; (3) abatement of £160 (instead of £120) when total income does not exceed £300, to which, we assume, the same remark applies; (4) a specially favourable way of applying abatements; and (5) option to be assessed on the actual income of the year, instead of any average.

AMOUNT TAXED.

Referring again to the detailed illustration figured above, we said that the £690 fixed what the rates were to be. But quite another matter is the question of the sum on which those rates are to be charged. No doubt the income is £690, but these two things have to be noted—

(*a*) When the practitioner pays the £410 he deducts tax at 6s., amounting in all to £123. Thus he has that money in hand as collector for the Revenue. In order to relieve him of it he is charged on the £410, and not at his own rate of tax, but at the full 6s. rate, which he deducts when he makes the payment.

(*b*) As to his own share, he is entitled to various tax abatements, all detailed above. They amount *in toto* to £290. That being taken off his income of £690 leaves, as we have seen, only £400 taxable at his rates. Thus in the net result he is to be taxed on £410 + £400, together £810. This is made up thus:—

| | | | |
|---|---|---|---|
| 1. Legal burdens charged at 6s. ... ... ... | | | £410 |
| 2. House, over and above ground burdens and mortgage interest. In this case it so happens that it is ... ... ... ... ... ... | | | nil |
| 3. Investment income at 3s. 9d. ... ... ... | | | 250 |
| 4. Professional income ... ... | | £800 | |
| Less | | | |
| Interest on price ... | £60 | | |
| Annuity ... ... | 300 | | |
| Tax abatements ... | 290 | | |
| | | 650 | |
| Charged at 3s. | | | £150 |
| As before ... ... | | | £810 |

The ways in which probably he will pay the tax will be these:—

1. *House.*—He will be assessed direct at 6s. on the No relief claimable, for it all comes off the ground burden and mortgage interest. £50
2. *Investment Income.*—It is assumed that this has all been taxed at the source at 6s. If so, 2s. 3d. per £ on the £250 (which is £28 2s. 6d.) will be allowed off No. 3 below. Or if received gross, then the direct assessment will be at the correct rate of 3s. 9d. This accounts for ... 250
3. *Professional Income.*—The direct Schedule D assessment will be—
   (1) Interest and annuity at 6s. £360
   (2) £800, less this £360 and less the abatements £290, leaves (taxable at 3s.) as above ... 150
   510

As before ... ... £810

